# Constitutively Active Signaling by the G Protein βγ-Subunit Mediates Intrinsically Increased Phosphodiesterase-4 Activity in Human Asthmatic Airway Smooth Muscle Cells

**DOI:** 10.1371/journal.pone.0118712

**Published:** 2015-03-05

**Authors:** Aihua Hu, Barry L. Diener, Maureen B. Josephson, Michael M. Grunstein

**Affiliations:** 1 Children’s Hospital of Philadelphia Research Institute, Philadelphia, Pennsylvania, United States of America; 2 University of Pennsylvania Perelman School of Medicine, Philadelphia, Pennsylvania, United States of America; SUNY College of Nanoscale Science and Engineering, UNITED STATES

## Abstract

Signaling by the Gβγ subunit of Gi protein, leading to downstream c-Src-induced activation of the Ras/c-Raf1/MEK-ERK1/2 signaling pathway and its upregulation of phosphodiesterase-4 (PDE4) activity, was recently shown to mediate the heightened contractility in proasthmatic sensitized isolated airway smooth muscle (ASM), as well as allergen-induced airway hyperresponsiveness and inflammation in an *in vivo* animal model of allergic asthma. This study investigated whether cultured human ASM (HASM) cells derived from asthmatic donor lungs exhibit constitutively increased PDE activity that is attributed to intrinsically upregulated Gβγ signaling coupled to c-Src activation of the Ras/MEK/ERK1/2 cascade. We show that, relative to normal cells, asthmatic HASM cells constitutively exhibit markedly increased intrinsic PDE4 activity coupled to heightened Gβγ-regulated phosphorylation of c-Src and ERK1/2, and direct co-localization of the latter with the PDE4D isoform. These signaling events and their induction of heightened PDE activity are acutely suppressed by treating asthmatic HASM cells with a Gβγ inhibitor. Importantly, along with increased Gβγ activation, asthmatic HASM cells also exhibit constitutively increased direct binding of the small Rap1 GTPase-activating protein, Rap1GAP, to the α-subunit of Gi protein, which serves to cooperatively facilitate Ras activation and, thereby, enable enhanced Gβγ-regulated ERK1/2-stimulated PDE activity. Collectively, these data are the first to identify that intrinsically increased signaling via the Gβγ subunit, facilitated by Rap1GAP recruitment to the α-subunit, mediates the constitutively increased PDE4 activity detected in asthmatic HASM cells. These new findings support the notion that interventions targeted at suppressing Gβγ signaling may lead to novel approaches to treat asthma.

## Introduction

Heterotrimeric G proteins play crucial roles in regulating the asthmatic state, including the induction of airway hyperresponsiveness (AHR) and inflammation [1]. Upon activation by G protein-coupled receptors (GPCRs) responding to a host of bronchoactive and proinflammatory stimuli, the G protein α subunit undergoes an exchange of GDP for GTP and becomes dissociated from the βγ subunits [2], thereby allowing both free Gα and Gβγ to activate their respective effectors, notably including those that stimulate the MAPK signaling pathways. The latter regulate various aspects of the airway asthmatic response including immune and inflammatory cell functions [3], as well as airway smooth muscle (ASM) function, due to activation of transcription factors and other downstream molecules that mediate the release of proinflammatory cytokines, chemokines, and other molecules that can alter ASM contractility and proliferation [4–7]. In this regard, GPCR-dependent (also receptor-independent) stimulation of the Ras/c-Raf1/MEK signaling cascade leading to downstream activation of the MAPK, ERK1/2, characteristically uses signals generated by the βγ subunits of the pertussis toxin (PTX)-sensitive family of G proteins that inhibits adenylate cyclase activity (i.e., Gi proteins) via activation of the tyrosine kinase, c-Src [8–12]. This PTX-sensitive Gi protein-regulated mechanism was found to play a particularly important role in mediating the heightened constrictor and impaired relaxation responses exhibited in isolated ASM tissues exposed to various proasthmatic conditions including passive sensitization with serum from atopic asthmatic patients [13], proinflammatory cytokine exposure [14], inoculation with rhinovirus [15], and prolonged heterologous and homologous β2-adrenergic receptor (β2AR) desensitization [16,17]. In this connection, the altered responsiveness exhibited in β2AR-desensitized ASM was attributed to upregulated phosphodiesterase 4 (PDE4) activity induced by activation of the Gβγ subunit of Gi protein and its consequent activation of c-Src-induced signaling via the Ras/c-Raf1/MEK pathway leading to ERK1/2 activation, the latter eliciting transcriptional upregulation of the PDE4D5 subtype [16,17].

Recently, the above Gi-βγ-regulated mechanism implicated in mediating PDE4-dependent proasthmatic changes in contractility in β2AR-desensitized ASM was also found to mediate the *in vivo* airway hyperresponsiveness and inflammation elicited by inhaled antigen challenge in a rabbit model of allergic asthma [18]. In light of this evidence, together with recent studies demonstrating a pivotal role for PDE4 activity in regulating airway function in asthmatic individuals [19–21] and in animal models of allergic asthma [22–26], and that PDE4 activity is intrinsically increased in cultured human ASM (HASM) cells isolated from asthmatic individuals [27], the present study sought to determine whether asthmatic HASM cells exhibit constitutively increased PDE activity that is mechanistically attributed to intrinsically upregulated Gβγ signaling coupled to c-Src-induced activation of the Ras/MEK/ERK1/2 pathway. The results demonstrated that: 1) relative to normal (non-asthmatic) HASM cells, primary cultures of asthmatic HASM cells exhibit markedly increased constitutive PDE4 activity associated with free (activated) Gβγ-coupled c-Src and ERK1/2 activation; 2) this Gβγ-regulated increase in PDE activity is associated with intrinsically enhanced co-localization of phosphorylated ERK1/2 with the PDE isoform, PDE4D, and 3) inhibition of Gβγ signaling acutely suppresses (within minutes) the increased PDE activity in asthmatic HASM cells to near normal levels, along with suppression of c-Src and ERK1/2 activation and co-localization of the latter with PDE4D. Finally, together with increased PDE activity attributed to free Gβγ-regulated ERK1/2 activation, the results demonstrated that asthmatic HASM cells also exhibit markedly increased direct binding of the small Rap1 GTPase-activating protein (Rap1GAP) to the α-subunit of G protein, a phenomenon that serves to cooperatively facilitate Ras-induced ERK1/2 activation, thereby enabling enhanced Gβγ-regulated PDE activity. Taken together, these new findings are the first to demonstrate that asthmatic HASM cells exhibit constitutively increased PDE activity that is mechanistically attributed to intrinsically increased Gβγ signaling, facilitated by Rap1GAP recruitment to the Gα-subunit, leading to heightened c-Src-dependent/Ras-mediated activation of ERK1/2 and its consequent direct binding to and accompanied activation of PDE4. Given the crucial role attributed to upregulated PDE activity in the pathobiology of asthma, these new findings highlight a heretofore-unidentified decisive role for Gβγ signaling in regulating the heightened PDE4 activity that characterizes the asthmatic airway, and suggest that interventions targeted at suppressing Gβγ signaling associated with Gi protein activation may lead to novel approaches to treat asthma.

## Methods

Normal and asthmatic isolated and cultured airway smooth muscle cells purchased from Lonza Walkersville, Inc. were used in this study, which was approved by the Committees for Protection of Human Subjects, Institutional Review Boards of the Children’s Hospital of Philadelphia.

### Materials

All chemicals were purchased from Sigma-Aldrich unless otherwise indicated.

### Culture and treatment of HASM cells

Primary cultures of HASM cells derived from bronchial smooth muscle freshly isolated post-mortem from normal non-asthmatic (n = 3) and asthmatic (n = 4) donor lungs were purchased at passage 2–3 from Lonza Walkersville, Inc. Other than data regarding age, race, and gender of the donors (see S1 Table), limited information was available with respect to clinical severity of asthma, treatment history, or cause of death, with exception to asthma donor #1. All the experiments were conducted at passage 5–6, after growing the cells in Lonza’s serum-containing SmBM medium supplemented with the BulletKit medium that contains insulin, human FGF, gentamycin/amphotericin B. The cells were maintained throughout in a humidified incubator containing 5% CO2 in air at 37°C and, after attaining ~90% confluence, cells were growth arrested for 24 hr in serum-free Ham’s F12 medium. Thereafter, the cells were examined for constitutive and induced changes in PDE activity, levels of free Gβγ, c-Src and ERK1/2 phosphorylation, and PDE4D protein expression, all in the absence and presence of different pretreatment conditions, as described.

### Assay of cAMP PDE activity

Levels of total cAMP PDE activity were determined in normal and asthmatic HASM cells at baseline and at different times ranging from 0.25–24 hr following administration of a maximally effective concentration of either: 1) a cell permeable anti-Gβγ blocking peptide (1 μM; AnaSpec; Premont, CA), comprised of a membrane permeable sequence (MPS; 15 amino acids) conjugated to the Gβγ-sequestering C-terminal domain (28 amino acids) of phosducin-like protein [28]; 2) the inert MPS peptide alone (1 μM) serving as a negative-control; or 3) 1μM of gallein [29], a recently described small molecule inhibitor of Gβγ signaling [30] (Acros Organics). PDE activity was also assayed in normal and asthmatic HASM cells following dose-dependent administration of anti-Gβγ blocking peptide (0.01–10 μM), and following treatment with various small molecule inhibitors, as described. The PDE activity assay was performed in the HASM cell lysates using a colorimetric, non-radioactive enzymatic assay (Enzo Life Sciences; Plymouth Meeting, PA) as per the manufacturer’s protocol, with some modification of our previously described approach [16,17]. Specifically, to maximize the sensitivity and yield of the measured enzyme activity, we used materials including a cell lysis buffer completely free of phosphate, composed of 50 mM Tris, pH 7.5, 0.1 mM EDTA, 0.1 mM EGTA, 1 mM DTT and 0.2% NP-40. The measured levels of PDE activity were standardized to protein content in the cell extracts. Finally, PDE activity was also assayed in normal and asthmatic HASM cells following transfection with siRNA preparations targeted at suppressing specific signaling molecules, as described below.

### siRNA-mediated knockdown studies

Targeted protein suppression using siRNA preparations was examined in normal and asthmatic HASM cells that were initially seeded into 6-well plates and, at ~40% confluency, the medium was replaced with the reduced serum-containing medium, Opti-MEM (Invitrogen). Cells were then transfected twice during a 24-hr interval with pools of siRNA preparations, each pool comprised of three siRNA duplexes targeted against either human Rap1GAP (Ambion; ID: s11785, s11786, s11787), RGS14, (Ambion; ID: 18059, 18147, 135857), or the PKAα catalytic subunit (Santa Cruz Biotechnology; Pool ID: sc-36240), as well as a non-targeted (scrambled) siRNA duplex serving as a negative control. As previously described [16,17,31], the transfections were carried out using Oligofectamine (Invitrogen) as the transfection agent, and the pools of siRNAs were applied to each well at a final concentration of 100 nM for each siRNA preparation. Consistent with our previous reports [16,17,31], this double-transfection approach enabled high transfection efficiency and, as detected by Western blotting, markedly inhibited expression of the targeted proteins by their respective siRNA preparations, with maximal inhibition detected at 72 hr following siRNA transfection averaging 82, 89, and 92% for RGS14, Rap1GAP and PKA proteins, respectively.

### Immunoblot analysis of Gβγ-regulated c-Src and ERK1/2 phosphorylation

Levels of total and phosphorylated c-Src (at residue Tyr416) and ERK1/2 proteins, as well as Gβ protein, were detected by Western blot analysis of lysates isolated from normal and asthmatic HASM cells before and at various times after treatment either with 1 μM of anti-Gβγ blocking peptide or gallein, or the c-Src family tyrosine kinase-selective inhibitor, SU6656 (10 μM), or exposure to the pro-asthmatic Th2 cytokine, IL-13 (50 ng/ml), as described. Following protein extraction and addition of gel loading buffer, the cellular extracts were loaded on a 10% SDS-PAGE gel for immunoblotting after transfer to a PVDF membrane. The membranes were then incubated overnight with monoclonal mouse anti-human primary antibodies directed against c-Src, phospho-c-Src^Tyr416^, ERK1/2 and phospho-ERK1/2 (Millipore; Billerica, MA), and a rabbit anti-human polyclonal antibody directed against the Gβ subunit (Millipore; Billerica, MA). Protein levels were detected by ECL after a 1-hr incubation with a 1:2,000 dilution of HRP-conjugated secondary antibody, followed by exposure to autoradiography film. The protein band intensities were quantified by densitometry.

### Co-immunoprecipitation studies

Normal and asthmatic HASM cells were prepared for co-immunoprecipitation (Co-IP) studies, performed as previously described [18], under native conditions in order to preserve protein:protein associations. After indicated treatments, cells were harvested and then exposed to lysis buffer. 1.5 mg of Protein G Dynabeads (Invitrogen; Carlsbad, CA) were incubated with 10 μg of anti-Gβ rabbit IgG (Millipore) or anti-Gα (pan) rabbit polyclonal antibody (Millipore; Billerica, MA) for 10 min at room temperature with rotation. Following several washes, the bound bead/antibody complex was added to sample, mixed by pipetting, and incubated for 2 hr at 4°C with rotation. After incubation, the beads with immobilized proteins were collected by placing the tubes in a magnetic field (DynaMag-spin Magnet, Invitrogen) and the remaining supernatants were stored and concentrated to measure the free Gβ protein. Total lysates (input), purified protein complexes (bound Gβ protein) and supernatants (free Gβ protein) were resuspended in sample loading buffer, boiled for 5 min, and then loaded onto a tris-acetate SDS polyacrylamide gel and separated by electrophoresis. The precipitated immunocomplexes were analyzed by immunoblotting using either anti-Gβ, anti-phospho-c-Src^Tyr416^, anti-ERK1/2, or anti-PDE4D rabbit polyclonal antibody, or anti-Rap1GAP rabbit monoclonal IgG antibody (Millipore; Billerica, MA), and unbound Gβ was detected in the uncaptured protein fraction by immunoblotting using anti-Gβ antibody.

### Statistical analyses

Results are expressed as mean ± SE values. Comparisons between normal and asthmatic HASM cell treatment groups were made using the Student t test (2-tailed). A probability of < 0.05 was considered statistically significant. Statistical analyses were conducted by using the Prism computer program by Graph Pad Software Inc.

## Results

### Intrinsically increased PDE activity in asthmatic HASM cells is mediated by activated Gβγ signaling

**Fig 1:**
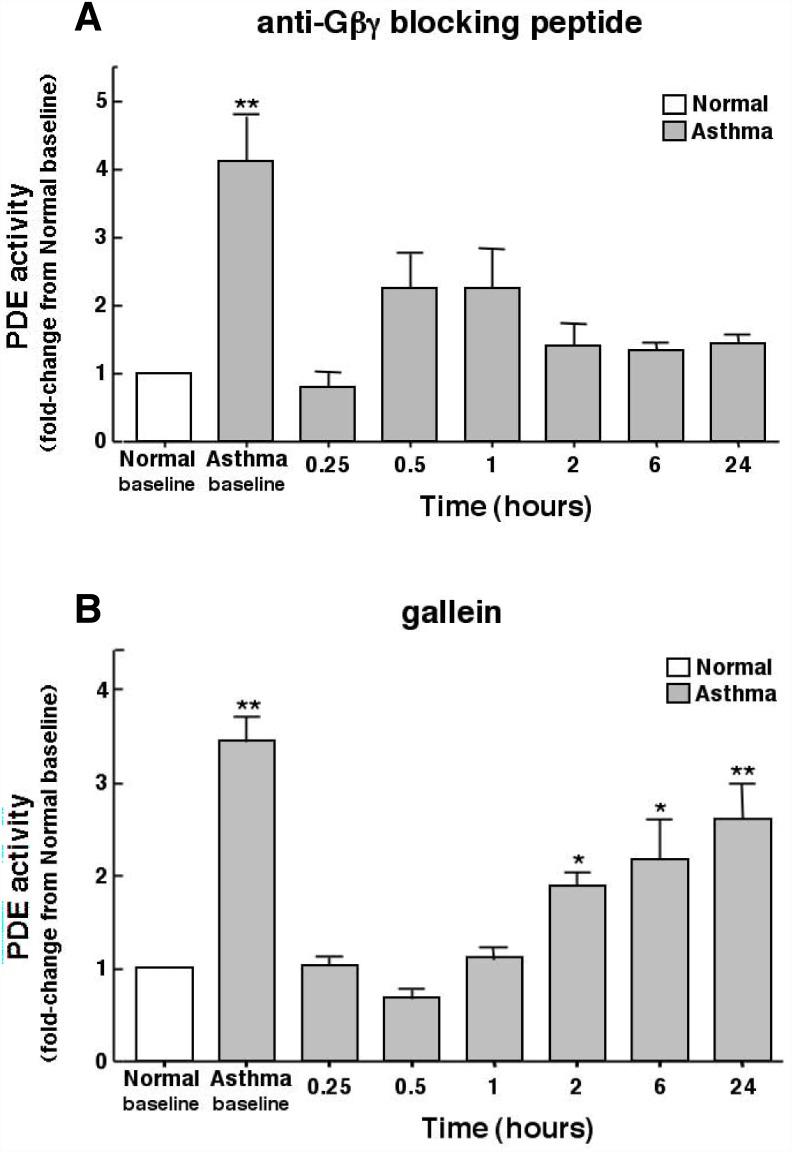
Inhibition of Gβγ signaling suppresses intrinsically increased PDE activity in asthmatic HASM cells: Comparison of suppressive effects of anti-Gβγ blocking peptide vs. gallein. Relative to normal cells, asthmatic HASM cells exhibit significantly increased constitutive (baseline) PDE activity. Inhibition of Gβγ signaling with a maximal effective concentration (1 μM) of either anti-Gβγ blocking peptide (**A**) or gallein (**B**) acutely suppresses PDE activity in asthmatic HASM cells to normal levels at 0.25 hr. Contrasting sustained inhibition of PDE activity in cells treated with anti-Gβγ blocking peptide, suppression of PDE activity in asthmatic HASM cells treated with gallein is transient, with progressive recovery in PDE activity after 1 hr that approaches near basal elevated levels by 24 hr. Data are mean ± SE values of 5–8 determinations made at each time point Comparisons are made using two-tailed Student t-test. *p<0.05; **p<0.01.

We previously implicated upregulated PDE4 activity due to activation of the βγ subunit of Gi protein in mediating airway constrictor hyperresponsiveness and inflammation in an *in vivo* rabbit model of allergic asthma [18], as well as the altered contractility elicited in isolated ASM tissues sensitized under various atopic-related and-unrelated proasthmatic conditions [16–18,32]. Given this evidence, together with that demonstrating a critical role for increased PDE4 activity in regulating airway function in asthmatic individuals [19–21], and that PDE4 activity is intrinsically increased in cultured asthmatic HASM cells [27], we hypothesized that asthmatic HASM cells exhibit constitutively increased PDE4 activity that is mechanistically attributed to upregulated Gβγ signaling. Initial studies demonstrated that, relative to normal (non-asthmatic) HASM cells wherein PDE activity averaged 62.5 ± 9.1 pmol/min/mg protein, basal PDE activity was markedly increased by approximately four-fold in cultures of asthmatic HASM cells (Fig. 1A). Treatment of the asthmatic cells with a maximally effective concentration of the anti-Gβγ blocking (sequestering) peptide (1 μM), comprised of the C-terminal domain of PhLP conjugated to an inert membrane permeable carrier peptide (MPS) [28], acutely suppressed PDE activity within 15 min to levels comparable to those detected in normal HASM cells, and this suppression was largely sustained for up to 24 hr (Fig. 1A). PDE activity was similarly acutely suppressed in asthmatic HASM cells that were treated with gallein (1 μM), a small molecule inhibitor of Gβγ signaling [29,30] (Fig. 1B). Unlike the anti-Gβγ blocking peptide, however, the suppressive effect of gallein was transient in nature, with progressive recovery in PDE activity that approached the heightened basal levels by 24 hr.

**Fig 2:**
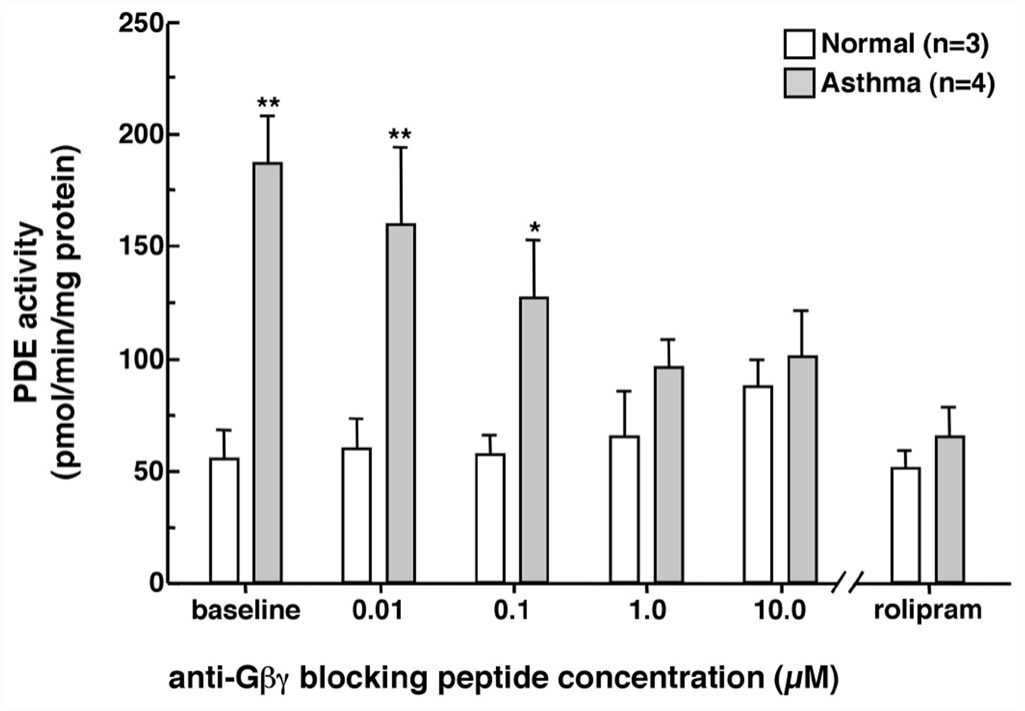
Dose-dependent effects of anti-Gβγ blocking peptide on PDE activity in normal vs. asthmatic HASM cells. Relative to normal cells, basal PDE activity is significantly increased in asthmatic HASM cells. Unlike in normal cells wherein administration of anti-Gβγ blocking peptide has no effect, asthmatic HASM cells treated with increasing doses of anti-Gβγ blocking peptide (each dose treatment for 6 hr) exhibit significant dose-dependent suppression of PDE activity. Note, the maximal suppressive effect attained using 1 μM is similar to that detected in asthmaic HASM cells treated with the PDE4 inhibitor, rolipram. Data are mean ± SE values. Comparisons of asthmatic HASM cells relative to basal normal levels are made using two-tailed Student t-test. *p<0.05; **p<0.01.

Because of its sustained action, the dose-dependent effects of separate treatments (each lasting 6 hr) with increasing concentrations of the anti-Gβγ blocking peptide on PDE activity were next assessed in HASM cells isolated from 3 normal and 4 asthmatic individuals. Unlike in normal cells wherein PDE activity remained largely unaltered, asthmatic HASM cells exhibited dose-dependent suppression of PDE activity following administration of the anti-Gβγ blocking peptide (Fig. 2). Maximal inhibition of PDE activity to near normal levels was attained using 1 μM of the anti-Gβγ peptide, and no further suppressive effect was detected following administration of 10 μM. In relation to these observations, as further depicted, the results obtained in separate experiments demonstrated that the heightened constitutive PDE activity in asthmatic HASM cells was also completely abrogated following treatment with the selective PDE4 inhibitor, rolipram (10 μM x 1 hr), a finding consistent with our previously reported findings demonstrating that rolipram inhibits the increased PDE activity evoked in cultured normal HASM cells and isolated rabbit ASM tissues sensitized under various proasthmatic conditions [16–18]. Moreover, this finding concurs with the observations in a recent report demonstrating that the increased PDE activity detected in cultured asthmatic HASM cells is inhibited by treatment with other selective PDE4 inhibitors, including roflumilast and cilomilast [27]. Thus, the above results demonstrate that the intrinsically increased PDE activity exhibited in asthmatic HASM cells is attributed to Gβγ-regulated activation of the PDE4 subtype (see below).

### Gβγ-regulated c-Src-coupled ERK1/2 activation is intrinsically increased in asthmatic HASM cells

**Fig 3:**
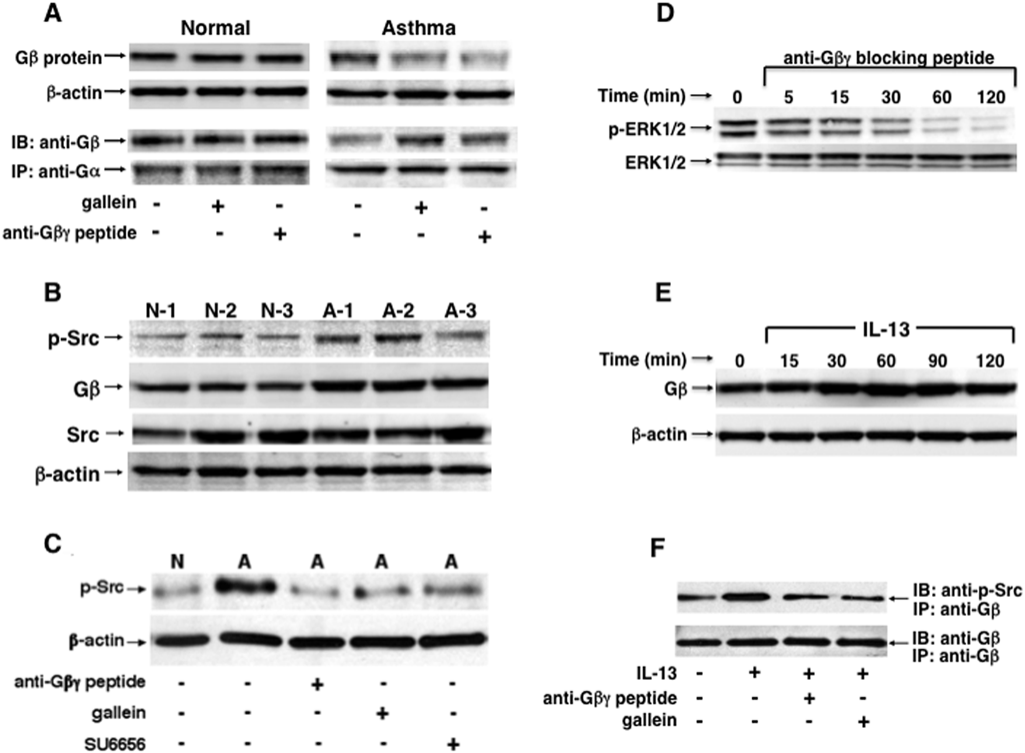
Gβγ-regulated c-Src and ERK1/2 activation is constitutively increased in asthmatic HASM cells and evoked in normal HASM cells stimulated with IL-13. (**A**) Western blot analysis demonstrating that, relative to normal cells, untreated asthmatic HASM cells exhibit constitutively increased free Gβ levels and correspondingly reduced co-localization of Gβ with immunoprecipitated Gα, denoting an intrinsic state of G protein activation. This pattern of Gβ distribution is acutely reversed in asthmatic HASM cells treated either with gallein or the anti-Gβγ blocking peptide (1 μM; x 30 min), By comparison, neither Gβγ inhibitor alters Gβ distribution in normal cells. (**B**) Immunoblots comparing 3 normal (N1–N3) and 3 asthmatic (A1–A3) HASM cell lines demonstrate that, under the same protein loading conditions yielding similar β-actin levels, constitutive expression of phosphorylated c-Src (p-Src) together with free Gβ protein are distinctly increased in the asthmatic HASM cell lines. Representative immunoblots in (**C**) and (**D**) demonstrate that, relative to normal cells, constitutively increased p-Src and p-ERK1/2 levels detected in asthmatic HASM cells, respectively, are acutely suppressed by treatment with either the anti-Gβγ blocking peptide or gallein (1 μM x 30 min), similar to the suppressive effect of treatment with the c-Src-selective inhibitor, SU6656. (**E**) Treatment of normal HASM cells with IL-13 (50 ng/ml) acutely evokes temporal increases in free Gβ protein levels, peaking at 60–90 min and remaining elevated above baseline at 120 min. (**F**) Co-IP experiment demonstrating that, relative to unstimulated cells, IL-13-treated (50 ng/ml x 60 min) normal HASM cells exhibit increased co-localization of p-Src with immunoprecipitated Gβ, and the latter is abrogated in IL-13-exposed HASM cells pretreated with either anti-Gβγ blocking peptide or gallein. The immunoblots shown in **A**-**F** are representative of 3–4 experiments.

In light of above results, a series of studies were conducted to systematically examine whether the intrinsically heightened Gβγ-regulated PDE activity in asthmatic HASM cells is mechanistically related to constitutively increased Gβγ activation associated with c-Src-induced ERK1/2 activation and its consequent acute stimulation of PDE activity. Accordingly, initial co-immunoprecipitation experiments compared intracellular levels of membrane-bound Gα-associated and-dissociated (“free”) Gβ, reflecting the inactive and activated states of G protein signaling, respectively, as well as levels of activated (phosphorylated) c-Src and ERK1/2 in lysates isolated from normal and asthmatic HASM cells under different treatment conditions. The representative immunoblots in Fig. 3A demonstrate that, under comparable protein loading conditions yielding similar β-actin levels, relative to normal cells, untreated asthmatic HASM cells exhibited constitutively increased free Gβ levels and, correspondingly, reduced co-localization of Gβ with immunoprecipitated Gα. Furthermore, this pattern of Gβ distribution, reflective of constitutively heightened Gβγ activation, was acutely reversed in asthmatic HASM cells that were treated either with gallein or the anti-Gβγ blocking peptide (1 μM), as evidenced by increased Gα-bound Gβ (Co-IP) and, correspondingly, reduced free Gβ levels detected at 30 min following treatment with either Gβγ inhibitor, whereas neither inhibitor had an effect in normal cells (Fig. 3A). Qualitatively similar results were obtained in two additional experiments and, based on densitometric quantification of the immunoblots, when correcting for any possible differences due to protein loading by analyzing changes in the ratio of free/Gα-bound (CO-IP) Gβ levels, the results demonstrated that, relative to untreated normal HASM cells (S1 Fig.): 1) normal cells treated with either Gβγ inhibitor showed no significant change in the ratio of Gβ free/Gα-bound; and 2) by comparison, the mean Gβ free/Gα-bound ratio was constitutively significantly increased in asthmaic HASM cells, and this heightened ratio was abrogated in asthmatic cells that were treated with either gallein or anti-Gβγ blocking peptide.

To ascertain whether the heightened state of constitutive Gβγ activation in asthmatic HASM cells is associated with altered downstream signaling events, we next compared the levels of c-Src and ERK1/2 activation in asthmatic vs. normal HASM cells, both in the absence and presence of Gβγ inhibition. Activated c-Src, denoted by its state of autophosphorylation at residue Tyr416 [33], and activated (phosphorylated) ERK1/2 were assessed using phospho-c-SrcTyr416- and phospho-ERK1/2-specific antibodies, respectively. Immunoblot analysis of these phosphorylated proteins in HASM cell lines isolated from 3 normal and 3 asthmatic individuals demonstrated that, under the same loading conditions yielding similar β-actin protein levels, relative to the normal (N) cells, the increased free Gβ protein levels detected in asthmatic (A) HASM cells were associated with increased levels of phosphorylated c-SrcTyr416 (p-Src), whereas total Src levels were comparable to those detected in normal cells (Fig. 3B). Moreover, in concert with its acute suppression of PDE activity, Gβγ inhibition with either the anti-Gβγ blocking peptide or gallein acutely suppressed (i.e., by 30 min) both the constitutively increased levels of: 1) p-Src detected in asthmatic HASM cells, similar to the suppressive effect of treatment with the c-Src family tyrosine kinase-selective inhibitor, SU6656 (Fig. 3C); and 2) phosphorylated ERK1/2 (p-ERK1/2), in accordance with the concept that the intrinsically increased Gβγ-regulated phosphorylation of c-Src is coupled to downstream activation of the Ras/c-Raf1/MEK-ERK1/2 pathway in asthmatic HASM cells (Fig. 3D).

Finally, to determine whether the constitutively heightened state of Gβγ-regulated signaling in asthmatic HASM cells parallels that which may be acutely induced by exogenous pro-asthmatic stimulation, naive normal HASM cells were exposed for varying durations to the pro-asthmatic Th2 cytokine, IL-13 (50 μg/ml), and then examined for induced activation of Gβγ and its direct coupling to phosphorylated c-Src. The immunoblot in Fig. 3E demonstrates that treatment with IL-13 acutely evoked temporal increases in free Gβ levels that peaked at 60–90 min and declined thereafter, but remained elevated above baseline at 120 min. Moreover, as depicted by a representative co-immunoprecipitation experiment in Fig. 3F, relative to unstimulated cells, enhanced co-localization of p-Src with immunoprecipitated Gβ was detected in HASM cells treated for 60 min with IL-13, and this induced association was abrogated in IL-13-exposed HASM cells that were pretreated with either the anti-Gβγ blocking peptide or gallein. It should be noted that these data were obtained under similar conditions of loading of immunoprecipitated Gβ, as demonstrated by the corresponding immunoblots using anti- Gβ antibody. Thus, these results support the concept that IL-13 acutely induces Gβγ activation that is accompanied by direct coupling of activated Gβγ with c-Src, a signaling event previously associated with acute ERK1/2 activation in IL-13-exposed HASM cells [18].

### Constitutively increased Gβγ-regulated c-Src-coupled ERK1/2 activation mediates intrinsically heightened PDE activity in asthmatic HASM cells

**Fig 4:**
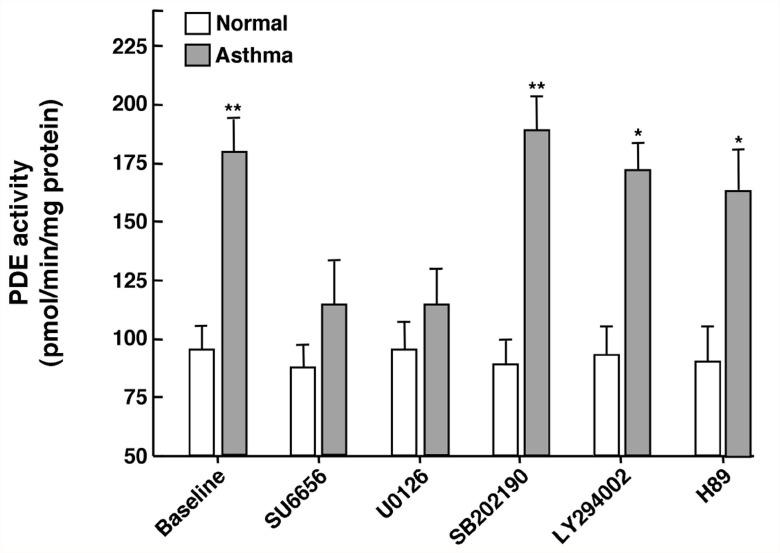
Comparison of effects of small molecule inhibitors of specific Gβγ-regulated downstream signaling proteins on PDE activity in asthmatic vs. normal HASM cells. Relative to baseline in normal HASM cells, constitutively heightened baseline PDE activity in asthmatic HASM cells is suppressed to near normal levels at 2 hr following treatment with either the c-Src inhibitor, SU6656, or the MEK-ERK1/2 inhibitor, U0126, whereas treatment with inhibitors of either p38 MAPK (SB202190), PI3K (LY294002), or PKA (H89) had no significant effect. Note: neither inhibitor had an appreciable effect on PDE activity in normal HASM cells. Data are mean ± SE values, each based on 3–6 determinations. Comparisons between asthmatic vs. normal HASM cells are made using two-tailed Student t-test. *p<0.05; **p<0.01.

Given the above results, we next investigated the role of the aforementioned Gβγ-regulated/Src-coupled ERK1/2 signaling mechanism, as well as other potential downstream molecules, in mediating the increased PDE activity detected in asthmatic HASM cells. Accordingly, asthmatic and normal HASM cell cultures were compared with respect to changes in PDE activity induced by treatment with previously reported maximally effective concentrations of specific inhibitors of signaling molecules potentially associated with Gβγ activation and upregulated PDE4 activity. As shown in Fig. 4, the significantly heightened basal PDE activity exhibited by untreated asthmatic HASM cells was acutely reversed to near normal levels following exposure (x 2 hr) to maximally effective concentrations of either the c-Src inhibitor, SU6656 (10 μM) or the MEK-ERK1/2 inhibitor, U0126 (5 μM), whereas treatment with inhibitors of either p38 MAPK (SB202190; 10 μM), PI3K (LY294002; 10 μM), or PKA (H89; 10 μM) had no significant effect. These data are consistent with the notion that the constitutively increased PDE activity in asthmatic HASM cells is largely attributed to a heightened state of Gβγ-regulated downstream signaling that involves increased c-Src-coupled ERK1/2 activation, rather than activation of the p38 MAPK, PI3K or PKA pathways.

**Fig 5:**
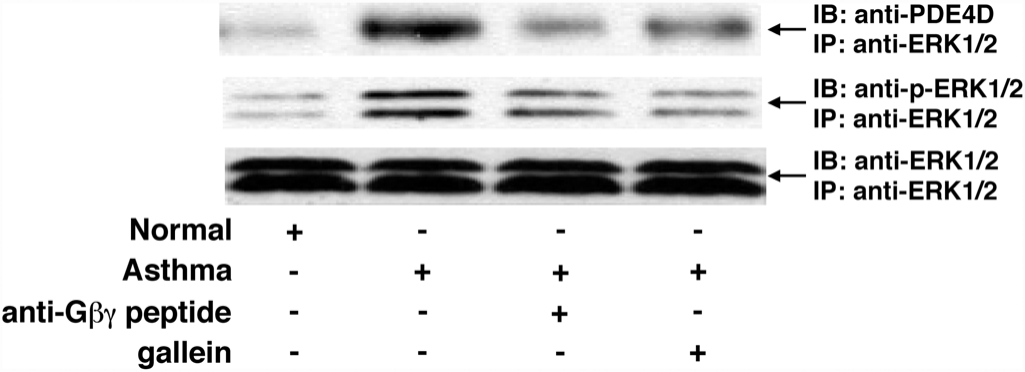
Inhibition of Gβγ signaling suppresses intrinsically increased direct coupling of p-ERK1/2 and PDE4D proteins in asthmatic HASM cells. Western blotting in Co-IP analysis demonstrating that, under the same protein loading conditions yielding similar β-actin levels, relative to normal cells, asthmatic HASM cells exhibit distinctly increased direct co-localization of immunoblotted (IB) PDE4D protein (apparent mol. wt. 100 kDa) with immunoprecipitated (IP) ERK1/2, the latter associated with heightened IB-detected p-ERK1/2. This heightened protein complex formation is acutely reversed (within 30 min) in asthmatic HASM cells treated with either anti-Gβγ blocking peptide or gallein. The immunoblots shown are representative of 3 experiments.

The above collection of data demonstrating that Gβγ inhibition can *acutely* (within minutes) reverse the increased PDE activity in asthmatic HASM cells raised the possibility that the enhanced PDE activity reflects a constitutively heightened state of Gβγ-regulated direct interaction between activated ERK1/2 and PDE4D, which reportedly represents the functionally relevant PDE4 isoform in HASM cells [34]. Indeed, such a direct interaction involving ERK1/2-induced phosphorylation and activation of PDE4D has been previously demonstrated accompanying protein kinase C stimulation in vascular smooth muscle cells [35]. Co-immunoprecipitation experiments addressing this possibility herein demonstrated that, under comparable protein loading conditions yielding similar immunoblotted levels of co-immunoprecipitated total ERK1/2, relative to normal cells, asthmatic HASM cells exhibited increased levels of co-immunoprecipitated p-ERK1/2, as well as increased co-immunoprecipitation of PDE4D with the ERK1/2 immunoprecipitate, whereas minimal or undetectable levels of such PDE4D co-localization was observed in normal cells (Fig. 5). Moreover, the heightened state of co-localization of ERK1/2 with PDE4D was acutely reversed (within 30 min) following treatment of asthmatic HASM cells with either the anti-Gβγ blocking peptide or gallein (Fig. 5). Thus, these data together with the above observations demonstrate that the constitutively increased Gβγ-regulated/ERK1/2-mediated PDE activity exhibited in asthmatic HASM cells (Figs. 3 and 4) is coupled to an intrinsically heightened state of direct interaction between activated ERK1/2 and PDE4D.

### Role of the GTPase-activating protein, Rap1GAP, in regulating PDE activity in asthmatic HASM cells

**Fig 6:**
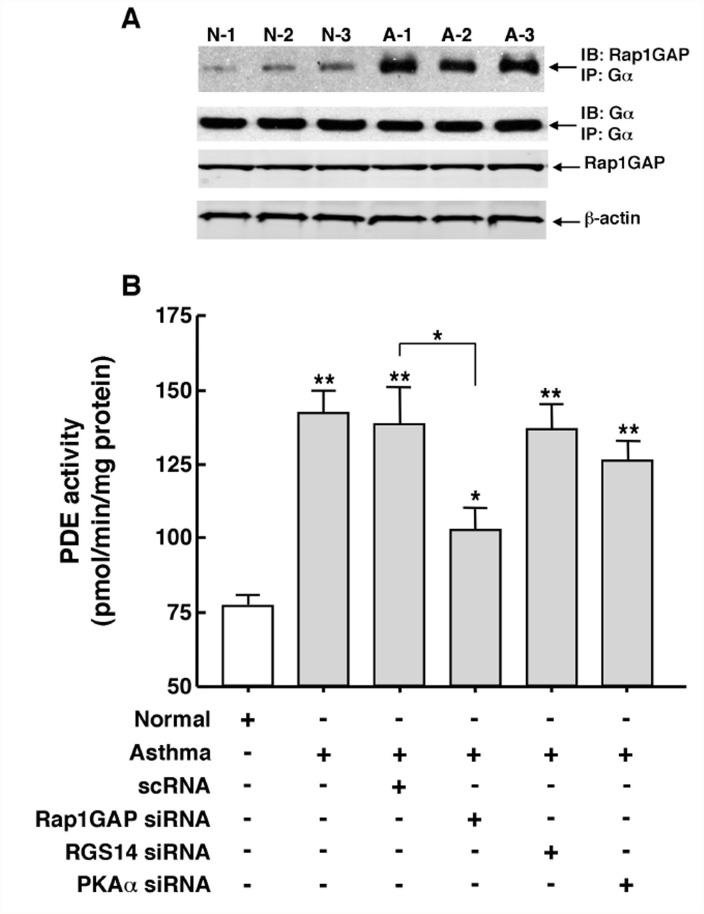
Constitutively increased co-localization of Rap1GAP with Gα protein contributes to upregulated PDE activity in asthmatic HASM cells. (**A**) Co-IP analysis demonstrating that, under comparable protein loading given by similar immunoblotted levels of co-immunoprecipitated Gα with anti-Gα immunoprecipitate, relative to 3 normal HASM cell lines, 3 asthmatic HASM cell lines exhibit markedly increased levels of co-immunoprecipitated Rap1GAP. Note: similar conditions of protein loading are also reflected by comparable immunoblotted levels of Rap1GAP, and β-actin. (**B**) Relative to normal HASM cells, intrinsically heightened PDE activity in asthmatic HASM cells is significantly suppressed by ~40% (p<0.05) in asthmatic cells transfected with a pool of siRNA duplexes targeted against Rap1GAP. By comparison, neither transfection with a scrambled (negative control) siRNA sequence (scRNA) nor pools of siRNA duplexes independently directed against RGS14 and PKAα had a significant effect on PDE activity in asthmatic HASM cells. Data are mean ± SE values, each based on 3–5 determinations. Comparisons between asthmatic vs. normal HASM cells, and between scRNA- vs. Rap1GAP siRNA-transfected asthmatic HASM cells, are made using two-tailed Student t-test. *p<0.05; **p<0.01. Note: neither of the siRNA preparations had a significant effect on PDE activity in normal HASM cells (data not shown).

Ras-induced downstream activation of ERK1/2 is inhibited by the Ras-related small GTP-binding protein, Rap1, suggesting that the latter may serve as an intrinsic homeostatic inhibitor of ERK1/2 activation [36–38]. Rap1 activity itself, however, is subject to suppression by the Rap1 GTPase, Rap1GAP (also Rap1GAPII), a phenomenon that arguably serves to facilitate Ras-induced downstream ERK1/2 activation [36–41]. In light of this evidence, we sought to determine the potential role of Rap1GAP in modulating the above-identified Gβγ-regulated signaling events implicated in mediating the intrinsically increased PDE activity in asthmatic HASM cells. In this regard, it is noteworthy that, apart from acting as a GTPase activating protein (GAP), Rap1GAP also serves as a guanine nucleotide dissociation inhibitor (GDI) that binds via its GoLoco amino acid motif to the α-subunit of the Gi family of heterotrimeric G-proteins [40,41]. Initial co-immunoprecipitation experiments demonstrated that asthmatic HASM cells exhibit constitutively increased co-localization of Rap1GAP with Gα. As shown in Fig. 6A, in comparing 3 normal and 3 asthmatic cells lines under similar protein loading conditions, evidenced by comparable immunoblotted levels of co-immunoprecipitated Gα with anti-Gα immunoprecipitate, the asthmatic HASM cells exhibited strikingly increased levels of co-immunoprecipitated Rap1GAP, whereas minimal such co-localization was detected in the normal cells. Given these observations, the potential role of Rap1GAP in regulating PDE activity was next examined by comparing the levels of PDE activity in cells transfected over 48 hr with pools of siRNA duplexes directed against either Rap1GAP, RGS14 (another GAP/GDI protein that also binds Gαi via its GoLoco motif. [42]), the catalytic α-subunit of protein kinase A (PKAα), or a scrambled siRNA (scRNA) sequence serving as a negative control. As shown in Fig. 6B, relative to normal cells, similar levels of significantly heightened PDE activity were detected in non-transfected and scRNA-transfected asthmatic HASM cells. This heightened activity was significantly suppressed by ~40% in asthmatic HASM cells that were transfected with the Rap1GAP siRNA duplexes, although the mean level of PDE activity expressed in these cells remained significantly greater than that detected in normal cells. By comparison, transfection with siRNAs directed against either RGS14 or PKAα had no significant effect on PDE activity in asthmatic HASM cells. Finally, it should be noted that neither of the siRNA preparations significantly affected PDE activity in normal HASM cells (data not shown). Collectively, these results are consistent with the notion that the heightened PDE activity detected in asthmatic HASM cells is partly, but significantly, attributed to constitutively increased co-localization of Rap1GAP with Gα, a phenomenon that is ostensibly permissive of Gi-βγ-stimulated Ras-induced ERK1/2 activation consequent to inactivation of Rap1 by Rap1GAP. In this context, our data suggest that the latter mechanism involving Rap1 suppression due to Rap1GAP binding to Gαi acts cooperatively with that involving activation of Ras through Gβγ signaling to transduce heightened activation of the Ras-stimulated c-Raf-MEK-ERK cascade mediating increased PDE activity in asthmatic HASM cells (see Discussion).

## Discussion

Increased PDE4 activity has been shown to play a decisive role in mediating the airway constrictor hyperresponsiveness evoked by allergen challenge in asthmatic individuals [19,21] and in animal models of allergic asthma [18,22–26], as well as the pro-asthmatic changes in contractility in isolated ASM tissue that accompanies its prolonged β2AR desensitization [16,17] or passive sensitization with atopic asthmatic serum or IL-13 [18]. The upregulated PDE4 activity was attributed to a mechanism that involves PTX-sensitive Gi-βγ activation under the different sensitizing conditions, which triggers c-Src-induced stimulation of the Ras/c-Raf/MEK signaling pathway leading to ERK1/2-dependent transcriptional upregulation of the PDE4D5 isotype and its consequent induction of the proasthmatic phenotype in the sensitized ASM [16–18,32]. In light of this evidence, the present study sought to determine whether asthmatic HASM cells exhibit constitutively increased PDE activity that is due to intrinsically upregulated signaling via the aforementioned Gβγ-regulated mechanism. The results are the first to demonstrate that: 1) relative to normal HASM cells, cultured asthmatic HASM cells intrinsically exhibit markedly increased rolipram-sensitive PDE activity that is regulated by constitutively increased free (activated) Gβγ-induced c-Src and ERK1/2 activation; 2) this heightened Gβγ-regulated ERK1/2-induced PDE activity involves enhanced direct co-localization of activated ERK1/2 with the PDE4D isoform in the asthmatic HASM cells; and 3) inhibition of this intrinsically heightened Gβγ-regulated signaling mechanism acutely suppresses PDE activity in asthmatic HASM cells to near normal levels, in association with suppression of c-Src and ERK1/2 activation and reversal of co-localization of ERK1/2 with PDE4D. Finally, together with intrinsically increased PDE activity attributed to free Gβγ-regulated ERK1/2 activation, the results demonstrated that asthmatic HASM cells also exhibit constitutively increased co-localization of Rap1GAP with the G protein α-subunit, a cooperative phenomenon that serves to facilitate Gβγ-regulated ERK1/2-induced activation of PDE activity in the asthmatic HASM cells (see below). Thus, the present data are the first to identify that asthmatic HASM cells exhibit constitutively enhanced PDE4 activity that is regulated by increased Gβγ signaling which, together with membrane recruitment of Rap1GAP, evokes heightened c-Src-stimulated downstream activation of ERK1/2 and its direct coupling to PDE4D. Given the important role attributed to increased PDE activity in the pathobiology of asthma, these new findings highlight a heretofore-unknown mechanism whereby constitutively increased Gβγ signaling regulates the altered phenotype in asthmatic HASM cells, suggesting that interventions targeted at suppressing this Gβγ-regulated signaling mechanism may enable novel approaches to treat asthma.

Among various issues raised by the present observations, the finding that asthmatic HASM cells exhibit constitutively increased PDE activity was not unexpected, given recent independent evidence demonstrating intrinsically heightened PDE4 activity in cultured asthmatic vs. normal HASM cells [27], and that treatment of asthmatic individuals with the PDE4 inhibitor, roflumilast, decreases airway hyperresponsiveness following allergen challenge [19,21] and improves lung function [20]. The observation that treatment with a Gβγ inhibitor *acutely* suppresses (within 15 min) this increased PDE activity (Fig. 1), however, was not anticipated, as we had previously demonstrated that the heightened PDE activity evoked in cultured normal HASM cells and naive isolated rabbit ASM tissues by prolonged exposure (i.e., ~24 hr) to various pro-asthmatic sensitizing conditions was due to transcriptional upregulation of PDE4D elicited by Gβγ-regulated ERK1/2 activation [16–18,32]. While such induced transcriptional control may also contribute to the increased PDE activity detected in asthmatic HASM cells, the observation that Gβγ inhibition acutely reverses this intrinsically increased PDE activity strongly suggests that another Gβγ-regulated signaling event(s) predominates in actively preserving the state of heightened PDE activity in asthmatic HASM cells, despite their maintenance in cell culture independent of any discernable persistent proinflammatory stimulation. This notion is supported by a series of present observations which demonstrated that, unlike in normal cells, asthmatic HASM cells exhibit constitutively increased Gβγ activity that acutely regulates Src and ERK1/2 activation (Fig. 3) which, in turn, mediates increased PDE activity (Fig. 4) accompanying direct coupling of activated ERK1/2 with PDE4D (Fig. 5). The latter finding concurs with those in an earlier study wherein, using comparably brief (<30 min) treatment regiments to exclude *de novo* protein synthesis, protein kinase C-induced stimulation of the Ras-c-Raf-MEK-ERK1/2 cascade was found to directly activate and translocate the membraneous PDE4D3 isoform in vascular smooth muscle cells [35]. Notwithstanding this consideration, the possibility that ERK1/2 activation may also acutely regulate PDE activity via various other mechanisms, including by altering PDE4D mRNA translation and/or PDE4D protein degradation, cannot be ruled out.

In contrast to our present observations that support the notion of a direct association between activated ERK1/2 and PDE4D which serves to stimulate PDE activity in asthmatic HASM cells, earlier studies by Houslay and colleagues (reviewed in [43]) demonstrated that ERK1/2 activation directly inhibits PDE4D5 activity in aortic smooth muscle cells. However, this group further reported that, due to resultant elevated cAMP levels leading to activation of PKA, the latter elicits phosphorylation of the PDE4 long isoform that acutely results in ablation of the inhibitory action of ERK1/2 phosphorylation, thereby implying “reprograming” of ERK1/2 from causing inhibition to net activation of PDE4D5 (43). While this scenario implicates an “indirect” role for ERK1/2 in stimulating PDE activity, it should be noted that our present observations demonstrated thattreatment with the ERK1/2 inhibitor, U0126, acutely suppressed the elevated PDE activity in asthmatic HASM cells to near normal levels, whereas neither treatment with the PKA inhibitor, H89 (Fig. 4), nor transfection with a previously reported effective pool of PKA-directed siRNA preparations [16] had an appreciable effect (Fig. 6). Thus, these data together with those demonstrating that asthmatic HASM cells exhibit intrinsically increased co-localization of activated ERK1/2 with PDE4D, and that this co-localization is acutely reversed by treatment with a Gβγ inhibitor (Fig. 5), suggest that Gβγ-regulated ERK1/2 activation plays a crucial role in directly inducing the increased PDE activity exhibited in asthmatic HASM cells. This evidence notwithstanding, a potential role for PKA activation and its modulation of ERK1/2-coupled regulation of PDE4 activity in asthmatic HASM cells cannot be completely ruled out.

While our present results are consistent with the prevailing concept that GPCR-dependent and receptor-independent stimulation of Ras-mediated ERK1/2 activation uses proximal signals generated by Gβγ-coupled c-Src activation [44–48], our data also concur with established evidence domonstrating that: 1) ERK1/2 activation is suppressed by recruitment of the GTP-binding protein, Rap1, which inhibits Ras function (36–38): and 2) this suppression is reversed by membrane recruitment of Rap1GAP, a GTPase which also acts as a GDI that binds via its GoLoco motif to the α-subunit of Gi protein, thereby inhibiting Rap1 activity [36–41]. Thus, whereas Rap1 activation may be viewed as a homeostatic “breaking” mechanism that counteracts Gβγ-coupled/c-Src-stimulated Ras activity, membrane recruitment of Rap1GAP serves to suppress the latter homeostatic action of Rap1 [39–41]. Accordingly, Gi protein activation leading to Gβγ-induced c-Src stimulation and Gα-coupled recruitment of Rap1GAP to inactivate Rap1 can be seen as acting cooperatively to heighten Ras stimulation of the MEK-ERK1/2 cascade, as previously proposed [39]. Our data herein support this notion, and further suggest that these Gi-regulated cooperative signaling events are constitutively activated in asthmatic HASM cells, given that the intrinsically heightened Gβγ-induced ERK1/2-stimulated PDE activity is abrogated by inhibition of c-Src signaling (Fig. 4), and is significantly attenuated by inhibition of Rap1GAP using targeted siRNA preparations (Fig. 6). In this context, it is of interest to note that activation of PKA in striatal neurons was recently shown to phosphorylate Rap1GAP and, thereby inhibit its GAP activity, resulting in an increase in Rap1 actvivty [49]. A priori, if also present in HASM cells, such an effect of PKA activation would be expected to attenuate Gβγ-regulated c-Src/Ras-mediated ERK1/2 activation. This raises the possibility that PKA activation (e.g., using cAMP-elevating agents) may act synergistically with an inhibitor of Gβγ-induced c-Src activation to suppress the pro-asthmatic state in asthmatic HASM cells. This consideration warrants systematic investigation.

In further considering the present observations, it should be noted that, unlike the sustained suppressive effect of treatment with anti-Gβγ blocking peptide on PDE activity in asthmatic HASM cells, the inhibitory effect of gallein was transient in nature (Fig. 1). Of interest, a disparity between these Gβγ inhibitors was also observed in our previous study wherein, unlike pretreatment with the anti-Gβγ blocking peptide which inhibited both the *in vivo* AHR and pulmonary inflammation elicited by allergen challenge in allergic rabbits, pretreatment with gallein only suppressed AHR and had no appreciable inhibitory effect on pulmonary inflammation (18). We speculated that, while the disparity between these inhibitors is not readily explained, possible explanations include differences in their pharmacodynamic or pharmacokinetic properties that might influence their inhibitory actions on Gβγ-regulated effector systems, and/or differences in their respective mechanisms of Gβγ inhibition [18]. Regarding the latter possibility, as previously reported [50], given that gallein is a small molecule with a distinctive spacial orientation of binding to the bioactive “hot spot” on the Gβγ surface, the nature and extent of its inhibition of Gβγ-targeted effector interactions may be relatively limited. Conversely, by sequestering the Gβγ subunit, the anti-Gβγ blocking peptide is arguably capable of relatively greater and more effective inhibition of Gβγ interactions with various effector targets [18]. These interesting considerations are worthy of systematic investigation.

In evaluating the implications of the present findings, it must be emphasized that the data pertain to studies conducted using a relatively small number of commercially available cultured HASM cell lines, with little history available regarding the asthmatic HASM cell donors (S1 Table). Thus, the extent to which our observations pertain to the *in vivo* human condition is open to speculation. In this regard, however, it is noteworthy that, notwithstanding the limited number of asthmatic cell lines examined, our results concur with those in previous studies, including the aforementioned study that also reported significantly increased intrinsic PDE4 activity in cultured HASM cells isolated from asthmatic individuals relative to normal non-asthmatic HASM cells [27], as well as the studies that implicated upregulated PDE4 actvity in mediating the *in vivo* airway responses to allergen challenge in asthmatic individuals [19,21] and in animal models of allergic asthma [18,22–26]. This concurance of findings is further substantiated when considering that the present observations are compatible with those in previous reports wherein Gβγ signaling coupled to ERK1/2 activation was shown to critically regulate the increased PDE4 activity experimentally induced in cultured normal HASM cells and isolated rabbit ASM tissues exposed to pro-asthmatic stimulation with either IL-13, atopic asthmatic serum, or long-acting β2AR agonists, as well as the corresponding PDE4-mediated pro-asthmatic changes in contractility exhibited by the stimulated ASM tissues [16–18,32]. In view of these considerations, we believe that the findings of the present study are likely applicable to the human asthmatic vs. normal *in vivo* condition.

In conclusion, this study is the first to report that intrinsically increased signaling by the Gβγ subunit, facilitated by recruitment of Rap1GAP to the Gαi-subunit, enables heightened Src-induced ERK1/2 activation and its consequent direct induction of the constitutively increased PDE4 activity exhibited in asthmatic HASM cells. To the extent that upregulated PDE4 activity is known to play a pivotal role in mediating the airway asthmatic phenotype, the present findings support the notion that future interventions targeted at suppressing Gβγ function may yield novel approaches to treat asthma.

## Supporting Information

S1 Table Demographic data for normal and asthmatic HASM cells. (TIF)

S1 Fig Intrinsically increased G protein activation in asthmatic HASM cells is reversed by inhibition of Gβγ signaling. Relative to normal cells, ratio of “free” (unbound)-to-Gα-bound Gβ levels (Gβ/Gβ:Gα-bound), determined by immunoblotting of non-complexed and complexed (co-immunoprecipitated) isolated fractions, respectively (see Methods), is significantly increased in asthmatic HASM cells, reflective of a heightened state of G protein activation. In contrast to normal cells, which show no effect, asthmatic HASM cells exhibit acute suppression of increased Gβ/Gβ:Gα-bound levels to near normal following treatment for 30 min with either gallein or anti-Gβγ blocking peptide. Data are mean±SE values based on 3–4 determinations under each treatment condition in n = 3 separate experiments. *p<0.05.
(TIF)

## References

[1] Druey KM. Regulation of G-protein-coupled signaling pathways in allergic inflammation. Immunol Res. 2009 ; 43 : 62 – 76. 10.1007/s12026-008-8050-0 18810336 PMC2687145

[2] Hepler JR, Gilman AG. G proteins. Trends Biochem Sci. 1992; 17: 383–387. 1455506 10.1016/0968-0004(92)90005-t

[3] Goldsmith ZB, Dhanasekaran DN. G protein regulation of MAPK networks. Oncogene 2007; 26: 3122–3142. 17496911 10.1038/sj.onc.1210407

[4] Gerthoffer WT, Singer CA. MAPK regulation of gene expression in airway smooth muscle. Respir Physiol Neurobiol. 2003; 137: 237–250. 14516729 10.1016/s1569-9048(03)00150-2

[5] Hakonarson H, Grunstein MM. Autocrine regulation of airway smooth muscle responsiveness. Respir Physiol Neurobiol. 2003; 137: 263–276. 14516731 10.1016/s1569-9048(03)00152-6

[6] Billington CK, Penn RB. Signaling and regulation of G protein-coupled receptors in airway smooth muscle. Respir Res. 2003; 4:2 Epub 2003: 1–28. 12648290 PMC152647

[7] Hershenson MB, Brown M, Camoretti-Mercado B, Solway J. Airway smooth muscle in asthma. Annu Rev Pathol Mech Dis. 2008; 3: 523–555. 18039134 10.1146/annurev.pathmechdis.1.110304.100213

[8] Stork PJ, Schmitt JM. Crosstalk between cAMP and MAP kinase signaling in the regulation of cell proliferation. Trends Cell Biol. 2002; 12:258–266. 12074885 10.1016/s0962-8924(02)02294-8

[9] Crespo P, Xu N, Simonds WF, Gutkind JS. Ras-dependent activation of MAP kinase pathway mediated by G-protein Gβγ subunits. Nature 1994; 369:418–420. 8196770 10.1038/369418a0

[10] Koch WJ, Hawes BE, Allen LF, Lefkowitz RJ. Direct evidence that Gi-coupled receptor stimulation of mitogen-activated protein kinase is mediated by Gβγ activation of p21ras. Proc Natl Acad Sci USA 1994; 91:12706–12710. 7809106 10.1073/pnas.91.26.12706 PMC45508

[11] Luttrell LM, Hawes BE, van Biesen T, Luttrell DK, Lansing TJ, Lefkowitz RJ. Role of c-Src tyrosine kinase in G protein-coupled receptor- and Gβγ subunit-mediated activation of mitogen-activated protein kinases. J Biol Chem. 1996; 271: 19443–19450. 8702633 10.1074/jbc.271.32.19443

[12] McCudden CR, Hains MD, Kimple RJ, Siderovski DP, Willard FS. G-protein signaling: back to the future. Cell Mol Life Sci. 2005; 62: 551–577. 15747061 10.1007/s00018-004-4462-3 PMC2794341

[13] Hakonarson H, Herrick DJ, Grunstein MM. Mechanism of impaired β-adrenoceptor responsiveness in atopic sensitized airway smooth muscle. Am J Physiol Lung Cell Mol Physiol. 1995; 269: L645–L652. 10.1152/ajplung.1995.269.5.L645 7491984

[14] Hakonarson H, Herrick DJ, Gonzalez Serrano P, Grunstein MM. Mechanism of cytokine-induced modulation of β-adrenoceptor responsiveness in airway smooth muscle. J Clin Invest. 1996; 97: 2593–2600. 8647953 10.1172/JCI118708 PMC507346

[15] Hakonarson H, Maskeri N, Carter C, Hodinka RL, Campbell D, Grunstein MM. Mechanism of rhinovirus-induced changes in airway smooth muscle responsiveness. J Clin Invest. 1998; 102: 1732–1741. 9802887 10.1172/JCI4141 PMC509121

[16] Hu A, Nino G, Grunstein JS, Fatma S, Grunstein MM. Prolonged heterologous β2-adrenoceptor desensitization promotes proasthmatic airway smooth muscle function via PKA/ERK1/2-mediated phosphodiesterase-4 induction. Am J Physiol Lung Cell Mol Physiol. 2008; 294:L1055–L1067. 10.1152/ajplung.00021.2008 18359889

[17] Nino G, Hu A, Grunstein JS, Grunstein MM. Mechanism regulating proasthmatic effects of prolonged homologous β2-adrenergic receptor desensitization in airway smooth muscle. Am J Physiol Lung Cell Mol Physiol. 2009; 297: L746–L757. 10.1152/ajplung.00079.2009 19666775 PMC2770790

[18] Nino G, Hu A, Grunstein JS, McDonough J, Kreiger PA, Josephson MB, et al G protein βγ-subunit signaling mediates airway hyperresponsiveness and inflammation in allergic asthma. PLoS One. 2012; 7(2): Epub 2012. 10.1371/journal.pone.0032078 PMC3284547 22384144

[19] van Schalkwyk E, Strydom K, Williams Z, Venter L, Leichtl S, Schmid-Wirlitsch C, et al Roflumilast, an oral, once-daily phosphodiesterase 4 inhibitor, attenuates allergen-induced asthmatic reactions. J Allergy Clin Immunol. 2005; 116: 292–298. 16083782 10.1016/j.jaci.2005.04.023

[20] Bateman ED, Izquierdo JL, Harnest U, Hofbauer P, Magyar P, Schmid-Wirlitsch C, et al Efficacy and safety of roflumilast in the treatment of asthma. Ann Allergy Asthma Immunol. 2006; 96: 679–686. 16729780 10.1016/S1081-1206(10)61065-4

[21] Louw C, Williams Z, Venter L, Leichtl S, Schmid-Wirlitsch C, Bredenbroker D, et al Roflumilast, a phosphodiesterase 4 inhibitor, reduces airway hyperresponsive- ness after allergen challenge. Respiration 2007; 74: 411–417. 16954654 10.1159/000095677

[22] Hansen G, Jin S, Umetsu DT, Conti M. Absence of muscarinic cholinergic airway responses in mice deficient in the cyclic nucleotide phosphodiesterase PDE4D. Proc Natl Acad Sci U S A 2000; 97: 6751–6756. 10841571 10.1073/pnas.97.12.6751 PMC18727

[23] Kanehiro A, Ikemura T, Makela MJ, Lahn M, Joetham A, Dakhama A, et al Inhibition of phosphodiesterase 4 attenuates airway hyperresponsiveness and airway inflammation in a model of secondary allergen challenge. Am J Respir Crit Care Med. 2001; 163: 173–184. 11208644 10.1164/ajrccm.163.1.2001118

[24] Tang HF, Song YH, Chen JC, Chen JQ, Wang P. Upregulation of phosphodiesterase-4 in the lung of allergic rats. Am J Respir Crit Care Med. 2005; 171:823–828. 15665325 10.1164/rccm.200406-771OC

[25] Sun JG, Deng YM, Wu X, Tang HF, Deng JF, Chen JQ, et al Inhibition of phosphodiesterase activity, airway inflammation and hyperresponsiveness by PDE4 inhibitor and glucocorticoid in a murine model of allergic asthma. Life Sci. 2006; 79: 2077–2085. 16875702 10.1016/j.lfs.2006.07.001

[26] Chapman RW, House A, Jones H, Richard J, Celly C, Prelusky D, et al Effect of inhaled roflumilast on the prevention and resolution of allergen-induced late phase airflow obstruction in Brown Norway rats. Eur J Pharmacol. 2007; 571: 215–221. 17610865 10.1016/j.ejphar.2007.05.074

[27] Trian T, Burgess JK, Niimi K, Moir LM, Ge Q, Berger P, et al β2-Agonist induced cAMP is decreased in asthmatic airway smooth muscle due to increased PDE4D. PLoS One. 2011; 6(5):e20000 10.1371/journal.pone.0020000 21611147 PMC3096656

[28] Chang M, Zhang L, Tam JP, Sanders-Bush E. Dissecting G protein- coupled receptor signaling pathways with membrane-permeable blocking peptides. J Biol Chem. 2000; 275: 7021–7029. 10702266 10.1074/jbc.275.10.7021

[29] Casey LM, Pistner AR, Belmonte SL, Migdalovich D, Stolpnik O, Nwakanma FE, et al Small molecule disruption of Gβγ signaling inhibits the progression of heart failure. Circ Res. 2010; 107: 532–539. 10.1161/CIRCRESAHA.110.217075 20576935 PMC2924955

[30] Lehmann DM, Seneviratne AM, Smrcka AV. Small molecule disruption of G protein βγ subunit signaling inhibits neutrophil chemotaxis and inflammation. Mol Pharmacol. 2008; 73:410–418. 18006643 10.1124/mol.107.041780 PMC2742223

[31] Hu A, Fatma S, Cao J, Grunstein JS, Nino G, Grumbach Y, et al Th2 cytokine-induced upregulation of 11beta-hydroxysteroid dehydrogenase-1 facilitates glucocorticoid suppression of proasthmatic airway smooth muscle function. Am J Physiol Lung Cell Mol Physiol. 2009; 296: L790–803. 10.1152/ajplung.90572.2008 19251840 PMC2681349

[32] Nino G, Hu A, Grunstein JS, Grunstein MM. Mechanism of glucocorticoid protection of airway smooth muscle from proasthmatic effects of long-acting beta2-adrenoceptor agonist exposure. J Allergy Clin Immunol. 2010; 125:1020–1027. 10.1016/j.jaci.2010.02.007 20392484 PMC2866838

[33] Kmiecik TE, Shalloway D. Activation and suppression of pp60c-src transforming ability by mutation of Its primary sites of tyrosine phosphorylation. Cell 1987; 49: 65–73.\ 3103925 10.1016/0092-8674(87)90756-2

[34] Billington CK, Le Jeune IR, Young KW, Hall IP. A major functional role for phosphodiesterase 4D5 in human airway smooth muscle cells. Am J Respir Cell Mol Biol. 2008; 38: 1–7. 17673687 10.1165/rcmb.2007-0171OC

[35] Liu H, Maurice DH. Phosphorylation-mediated activation and translocation of the cyclic AMP-specific phosphodiesterase PDE4D3 by cyclic AMP-dependent protein kinase and mitogen-activated protein kinases. A potential mechanism allowing for the coordinated regulation of PDE4D activity and targeting. J Biol Chem. 1999; 274:10557–10565. 10187850 10.1074/jbc.274.15.10557

[36] Kitayama H. Sugimoto Y, Matsuzaki T, Ikawa Y, Noda M. A ras-related gene with transformation suppressor activity. Cell 1989; 56: 77–84. 2642744 10.1016/0092-8674(89)90985-9

[37] Cook SJ, Rubinfeld B, Albert I, McCormick F. RapV12 antagonizes Ras-dependent activation of ERK1 and ERK2 by LPA and EGF in Rat-1 fibroblasts. EMBO J. 1993; 12: 3475–3485. 8253074 10.1002/j.1460-2075.1993.tb06022.x PMC413624

[38] Okada S. Matsuda M, Anafi M, Pawson T, Pessin JE. Insulin regulates the dynamic balance between Ras and Rap1 signaling by coordinatingthe assembly states of the Grb2-SOS and CrkII-C3G complexes. EMBO J. 1998; 17: 2554–2565. 9564038 10.1093/emboj/17.9.2554 PMC1170597

[39] Mochizuki N, Ohba Y, Kiyokawa E, Kurata T, Murakami T, Ozaki T, et al Activation of the ERK/MAPK pathway by an isoform of rap1GAP associated with G alpha(i). Nature. 1999; 400: 891–894. 10476970 10.1038/23738

[40] Willard FS, Low AB, McCudden CR, Siderovski DP. Differential G-alpha interaction capacities of the GoLoco motifs in Rap GTPase activating proteins. Cell Signal. 2007; 19: 428–438. 16949794 10.1016/j.cellsig.2006.07.013

[41] van Dam TJ, Bos JL, Snel B. Evolution of the Ras-like small GTPases and their regulators. Small GTPases. 2011; 2: 4–16. 21686276 10.4161/sgtp.2.1.15113 PMC3116619

[42] Shu FJ, Ramineni S, Hepler JR. RGS14 is a multifunctional scaffold that integrates G protein and Ras/Raf MAPkinase signalling pathways. Cell Signal. 2010; 22: 366–376. 10.1016/j.cellsig.2009.10.005 19878719 PMC2795083

[43] Houslay MD, Adams DR. PDE4 cAMP phosphodiesterases: modular enzymes that orchestrate signalling cross-talk, desensitization and compartmentalization. Biochem J. 2003; 370: 1–18. 12444918 10.1042/BJ20021698 PMC1223165

[44] Stork PJ, Schmitt JM. Crosstalk between cAMP and MAP kinase signaling in the regulation of cell proliferation. Trends Cell Biol. 2002; 12:258–266. 12074885 10.1016/s0962-8924(02)02294-8

[45] Crespo P, Xu N, Simonds WF, Gutkind JS. Ras-dependent activation of MAP kinase pathway mediated by G-protein Gβγ subunits. Nature 1994; 369:418–420. 8196770 10.1038/369418a0

[46] Koch WJ, Hawes BE, Allen LF, Lefkowitz RJ. Direct evidence that Gi-coupled receptor stimulation of mitogen-activated protein kinase is mediated by Gβγ activation of p21ras. Proc Natl Acad Sci USA 1994; 91:12706–12710. 7809106 10.1073/pnas.91.26.12706 PMC45508

[47] Luttrell LM, Hawes BE, van Biesen T, Luttrell DK, Lansing TJ, Lefkowitz RJ. Role of c-Src tyrosine kinase in G protein-coupled receptor- and Gβγ subunit-mediated activation of mitogen-activated protein kinases. J Biol Chem. 1996; 271: 19443–19450. 8702633 10.1074/jbc.271.32.19443

[48] McCudden CR, Hains MD, Kimple RJ, Siderovski DP, Willard FS. G-protein signaling: back to the future. Cell Mol Life Sci. 2005; 62: 551–577. 15747061 10.1007/s00018-004-4462-3 PMC2794341

[49] Smrcka AV. G protein βγ subunits: Central mediators of G protein-coupled receptor signaling. Cell Mol Life Sci. 2008; 65:2191–2214. 10.1007/s00018-008-8006-5 18488142 PMC2688713

[50] McAvoy T, Zhou MM, Greengard P, Nairn AC. Phosphorylation of Rap1GAP, a striatally enriched protein, by protein kinase A controls Rap1 activity and dendritic spine morphology. Proc Natl Acad Sci U S A. 2009; 106:3531–3536. 10.1073/pnas.0813263106 19218462 PMC2651273

