# Supplementary figures and images for: Constitutively Active Signaling by the G Protein βγ-Subunit Mediates Intrinsically Increased Phosphodiesterase-4 Activity in Human Asthmatic Airway Smooth Muscle Cells
Source: PLoS One. 2015 Mar 5;10(3):e0118712. doi: 10.1371/journal.pone.0118712 (PMC4351001; doi:10.1371/journal.pone.0118712)

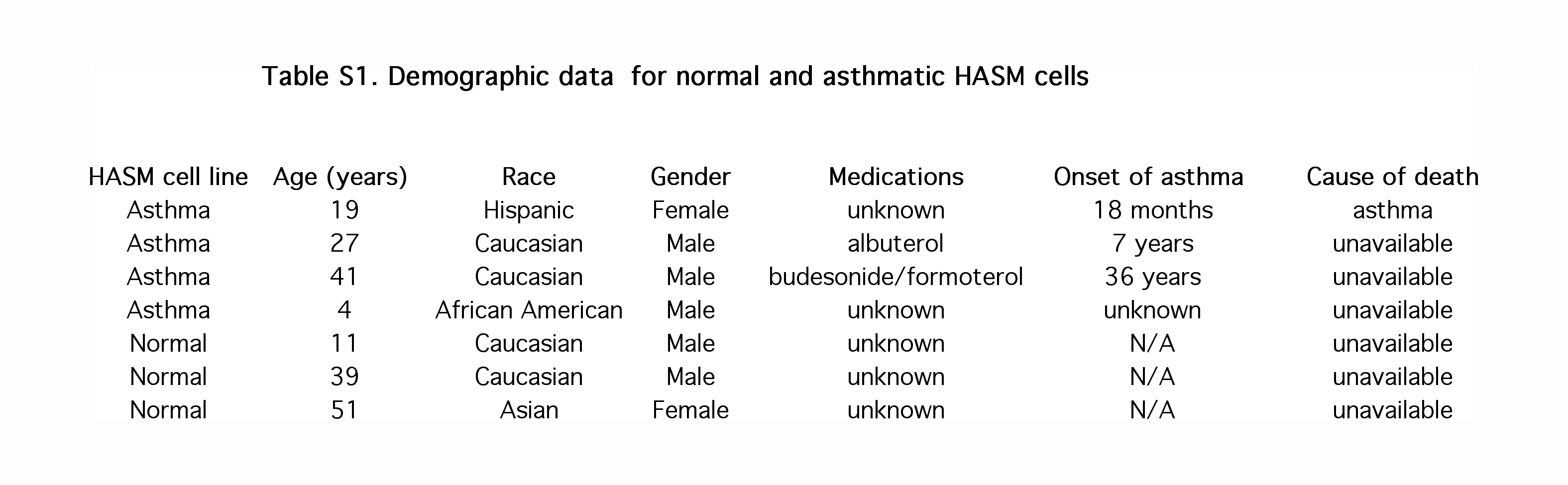

Supplement: S1 Table — (TIF) [file pone.0118712.s001.tif]

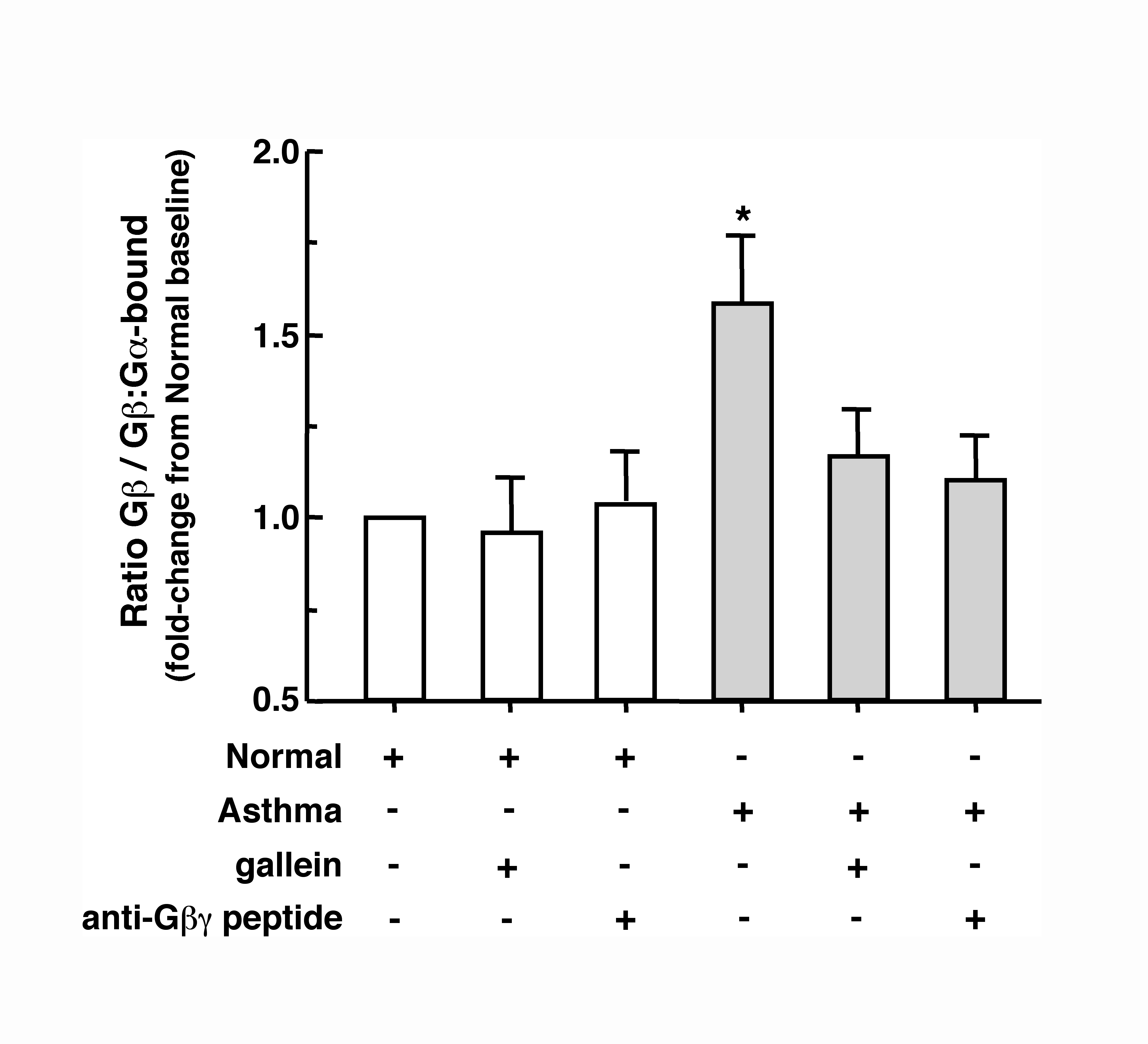

Supplement: S1 Fig — Relative to normal cells, ratio of “free” (unbound)-to-Gα-bound Gβ levels (Gβ/Gβ:Gα-bound), determined by immunoblotting of non-complexed and complexed (co-immunoprecipitated) isolated fractions, respectively (see Methods), is significantly increased in asthmatic HASM cells, reflective of a heightened state of G protein activation. In contrast to normal cells, which show no effect, asthmatic HASM cells exhibit acute suppression of increased Gβ/Gβ:Gα-bound levels to near normal following treatment for 30 min with either gallein or anti-Gβγ blocking peptide. Data are mean±SE values based on 3–4 determinations under each treatment condition in n = 3 separate experiments. *p<0.05. (TIF) [file pone.0118712.s002.tif]
